# False Alarms in Consumer Genomics Add to Public Fear and Potential Health Care Burden

**DOI:** 10.3390/jpm10040187

**Published:** 2020-10-23

**Authors:** Xiaoming Liu, Deborah Cragun, Jinyong Pang, Swamy R. Adapa, Renee Fonseca, Rays H. Y. Jiang

**Affiliations:** 1 College of Public Health, University of South Florida, Tampa, FL 33612, USA; DCragun@usf.edu (D.C.); jpang@usf.edu (J.P.); swamyrakesh@usf.edu (S.R.A.); Jiang2@usf.edu (R.H.Y.J.); 2 Morsani College of Medicine, University of South Florida, Tampa, FL 33612, USA; reneefonseca@mail.usf.edu

**Keywords:** direct to consumer genomics, microarray, false alarm, genotyping error, variant classification error

## Abstract

We have entered an era of direct-to-consumer (DTC) genomics. Patients have relayed many success stories of DTC genomics about finding causal mutations of genetic diseases before showing any symptoms and taking precautions. However, consumers may also take unnecessary medical actions based on false alarms of “pathogenic alleles”. The severity of this problem is not well known. Using publicly available data, we compared DTC microarray genotyping data with deep-sequencing data of 5 individuals and manually checked each inconsistently reported single nucleotide variants (SNVs). We estimated that, on average, a person would have ~5 “pathogenic” alleles reported due to wrongly reported genotypes if using a 23andMe genotyping microarray. We also found that the number of wrongly classified “pathogenic” alleles per person is at least as significant as those due to wrongly reported genotypes. We show that the scale of the false alarm problem could be large enough that the medical costs will become a burden to public health.

## 1. Introduction

As of 2019, over 26 million members of the public have undergone “direct-to-consumer” (DTC) genetic testing to discover more about their ancestry and/or determine what their DNA can reveal about their health, wellness, and unique traits [1]. Several popular consumer tests, such as 23andMe, AncestryDNA, and FamilyTreeDNA, use microarrays to assess thousands of single nucleotide variants (SNVs) across the genome. However, DTC companies typically interpret only a portion of the data. Nevertheless, the entire set of SNVs is available to customers who can upload the data online to obtain additional health information from third-party data interpretation services [2]. DTC results and raw data should be confirmed with a healthcare provider before making any medical decisions; a statement included in the disclaimers of DTC services. However, it is uncertain whether these precautionary messages are understood or are persuasive enough to be heeded by most consumers. Furthermore, it remains unclear whether actions taken by consumers or their healthcare providers in response to DTC results are medically appropriate.

Recent lines of evidence show that DTC results may add to the health care burden [3,4]. One publication describes several individuals who thought they or their child might have a potentially fatal genetic heart condition based on the results of DTC tests [3]. In some cases, surgery or other medical actions were taken before it was determined that the DTC test result was incorrect [3]. In some other cases, patients who did not undergo medical treatment experienced high anxiety and worry due to the severe and often deadly nature of the disease they believed they or their child had [3]. Those cases reports suggest a potential for psychological harm in addition to medical harms and a potential economic burden. Another article, based on a cohort of more than 1000 DTC participants, estimated that more than half of the consumers used at least one third-party tool for secondary genomic data interpretations [2]. Importantly, in another survey, half of the DTC participants reported discussing concerns about the DTC results with healthcare providers, and more than 10% of the participants made additional appointments exclusively based on DTC results or third-party interpretations [4].

The extent to which DTC tests burden our healthcare system is not clear. Two studies found that between 40% and 50% of patient samples received to verify DTC results contained false positives that could not be confirmed in a clinical laboratory [5,6]. However, those results were based on biased sampling, so did not show the accurate scale of the false positives for a random sample of the population. A separate study found that although the overall sensitivity, specificity, positive predictive value, and negative predictive value of the SNV-microarray evaluated all rate above 99%, the likelihood of a true positive was substantially reduced for low-frequency alleles [7]. A false discovery rate of over 84% for variants with a frequency of <0.001% was determined. Those authors concluded that SNV-microarrays are highly inaccurate for genotyping rare and clinically-actionable variants [7]. Although the SNV-microarray analyzed in that study was different from the SNV-microarrays used by the major DTC testing companies, the findings raise significant concerns. Consequently, it is critical to better understand the number of individuals undergoing DTC testing who could be negatively impacted by a false alarm.

## 2. Materials and Methods

Summary: We identified a total of 5 individuals from the Harvard Personal Genomics Project who have uploaded three types of genome data: DTC microarray genotyping results, whole genome sequencing results (files in Variant Call Format (VCF)), and raw sequencing data (files in Binary Alignment Map (BAM) format). We then distinguished two sources of false-positive errors, one due to errors of genotyping (incorrect test result), and the other due to incorrect variant classification. For the genotyping error, we delineated discrepancies between array and NGS results and used the raw sequence reads to determine the true genotypes. A total of 4979 discrepant genotypes were found, and the true genotypes were determined one by one manually. For the potential classification error, we evaluated variants listed as pathogenic in ClinVar and used the allele frequencies of these variants from worldwide human populations to identify those that are unlikely to be pathogenic given their relatively high allele frequency in any given population (>10%).

Sample Selection: Five individuals (hu85ADFB, hu9AF7CC, huC58DAE, huD3FFCB, huCDD5EE) were selected from the Harvard Personal Genomics Project as they include microarray genotyping data from 23andMe (hu85ADFB, hu9AF7CC, huC58DAE, huD3FFCB) or Family Tree DNA (huCDD5EE), and whole-genome sequencing data (VCF and BAM files) from Veritas Genetics [8].

Variant Annotation: Single-nucleotide variants (SNVs) were annotated with ClinVar [9] (downloaded 3 November 2019) and HGMD [10] (professional 2017.2) using the WGSA (WGS Annotator) pipeline [11]. Allele frequencies from various populations were obtained from the PGG.SNV database [12].

Genotype Error Estimation: Microarray genotypes were first compared with genotypes from the VCF file, and all chromosome positions reported in both files (N) were selected. All genotypes that disagreed with each other were manually checked by reviewing the corresponding reads from the BAM files using the integrative genomics viewer [13], and correct genotypes were determined. Assuming the reported genotypes are correct if microarray genotypes agree with sequence genotype, the number of reference alleles and alternative alleles in the correct genotypes of the N positions were counted as $N_{r}$ and $N_{a}$ respectively. Errors were separated into three types: reference alleles mistakenly called as alternative alleles ($n_{1}$), alternative alleles mistakenly called as other alternative alleles ($n_{2}$), and alternative alleles mistakenly called as reference alleles ($n_{3}$). The corresponding error rates were calculated as $r_{1}=n_{1}/N_{r}$, $r_{2}=n_{2}/N_{a}$, and $r_{3}=n_{3}/N_{a}$.

Expected Pathogenic Genotypes Due to Genotyping Error: Assuming all microarray genotypes that agree with genotypes reported in VCF files, and those not reported in the corresponding VCF files are correct, given the error rates $r_{1}=1\times 10^{-3}$ (the reference allele to an alternative allele), $r_{2}=6\times 10^{-5}$ (an alternative allele to another alternative allele), and $r_{3}=4\times 10^{-4}$ (an alternative allele to the reference allele), we can produce a list of all possible wrong genotypes with corresponding probabilities for all chromosome positions reported in the microarray data. The list of SNVs was annotated, and the summation of the probabilities of those genotypes annotated as Pathogenic or Likely Pathogenic by ClinVar were deemed the expected pathogenic genotypes due to genotyping error. These calculations were conducted for each individual separately.

## 3. Results

Here, we sought to estimate the average number of variants that could create false alarms in each DTC test result (Table 1). In our study, we classified false alarms as (1) an incorrect test result where the pathogenic (i.e., disease-causing) variant that was reported likely resulted from SNV-microarray genotyping errors and is not present in the raw sequencing data, or (2) incorrect classification whereby the variant is present but allele frequencies in worldwide populations suggest the effect is likely benign. Both of these concerns could result in a false alarm by leading someone to believe they had a genetic risk that they do not. Note while the first type of false alarm (due to reported incorrect genotypes) can be well defined, the second type (due to false classification) cannot be because we do not know whether the variant is truly pathogenic or benign without extensive experiments. Therefore, we will focus on the estimation of the first type and only discuss the scale of the second type.

To estimate the number of false alarms generated by DTC, we manually cross-validated independently generated array results and whole genome sequencing data from the same five individuals from the Harvard Personal Genomics Project. Among them, four individuals have 23andMe array genotyping data (2 have ~580,000 SNVs and 2 have ~930,000 SNVs), and the other individual has FamilyTreeDNA array data (688,413 SNVs). After comparing the array genotypes with the corresponding sequencing results (VCF files), 1,414,206 (263,802 to 445,043 per person) SNV genotypes were reported by both the array and VCF. Among them, a total of 4979 disagreements (363 to 2139 per person) were found between the array reports and sequencing reports (Table S1). We manually checked all 4979 disagreements by reviewing the corresponding raw sequencing data (BAM files). For genotyping errors in the array data, we found a reference allele mistakenly called an alternative allele every $1\times 10^{3}$ bases on average. An alternative allele was mistakenly called a different alternative allele every $6\times 10^{5}$ bases on average and an alternative allele was mistakenly called the reference allele every $4\times 10^{4}$ bases on average. Based on these three types of genotyping error rates, we estimate that, on average, an individual has 1286 erroneous alternative alleles called (652 to 2270 per person). The four individuals with 23 and Me array data have an average of 7.5 pathogenic SNVs that are wrongly called alternative alleles (i.e., false positives), and the individual with FamilyTreeDNA data has one false positive pathogenic SNV (Table 1, Table S1). Among the 31 total false positives, many are classified as “underlying severe diseases”. We then investigated the possibility that some of the observed pathogenic SNVs with correct genotypes may also be false alarms due to incorrect classifications in ClinVar. We found 21 such correctly genotyped pathogenic SNVs (average 7.5 for 23 and Me users and 3 for the FamilyTreeDNA user, respectively). Although it remains somewhat uncertain whether these variants are pathogenic, we can use allele frequencies to suggest that pathogenicity is unlikely because common variants are more likely to be benign while rare variants are more likely to be pathogenic [14]. Among these 21 instances, 16 “pathogenic alleles” have >10% allele frequencies in at least one worldwide population, suggesting that they are unlikely to be genuinely pathogenic (Table S2). Additionally, some of these 16 SNVs are classified as pathogenic for dire conditions such as colon carcinoma, ataxia-oculomotor apraxia type 1, hereditary prostate cancer, and congenital heart disease. We then used InterVar [15] to classify the 21 SNVs based on the ACMG/AMP 2015 guideline [16]. Nine SNVs were classified as benign and nine were unknown or classified as of uncertain significance (Table S2-1).

## 4. Discussion

As DTC tests boast over 99% accuracy, we are concerned that individuals undergoing DTC testing may not truly understand the gravity of using raw data to identify health risks. Our data found that all five individuals had inaccurate SNVs in genes associated with concerning medical conditions. Furthermore, several other SNVs were found that may also unnecessarily raise concerns about health conditions due to uncertainty in determining whether they are pathogenic or benign. Many individuals overestimate the utility of genetic information, as evidenced by a study where ~59% of respondents indicated that the information from DTC testing would influence the management of their health [17]. The reality is that even when DTC results are technically accurate, only a small portion of SNVs currently meet the following criteria to be clinically actionable: (1) the SNV interferes with gene function (i.e., is a pathogenic variant), (2) the pathogenic variant substantially increases disease risk (i.e., is highly penetrant), (3) identifying the pathogenic variant changes medical care (beyond what would already be recommended as part of healthy living), and (4) such changes in care are associated with improvements in health outcomes.

Understanding the nuances and variability in genetic testing methods and standards can be challenging. Genetic health data interpreted directly by DTC companies must meet specific FDA (U.S. Food and Drug Administration) standards for accuracy and clinical validity, but data provided for “wellness” purposes need not meet such standards. Furthermore, raw data are not subject to any FDA standards even though it contains health-related information, which may or may not be accurate, and raw data interpretations can be challenging to understand. For over a decade the National Society of Genetic Counselors (NSGC) has recommended consumers consider several issues before undergoing DTC testing [18]. NSGC also has a position statement cautioning about false-positive and false-negative data that raw data files may contain and the importance of confirming results in a clinical laboratory before using them to make health decisions [19]. As the misuse of DTC test results is directly related to the research and practice of personalized medicine, we appeal to a broader array of professional societies to actively engage in outlining recommendations for the practice.

Classification efforts taken to determine if a variant is pathogenic are also challenging and should not be made solely using easily accessible databases. Laboratories that conduct clinical genetic testing for healthcare purposes typically use sequencing technologies and follow American College of Medical Genetics (ACMG) recommendations when annotating variants, a process that requires multiple lines of evidence to be met before calling a variant “pathogenic” [16]. Our results also showed that following its guideline can indeed reduce the number of false alarms of the second type. Yet those complicated and conservative practices may not be fully fulfilled by third-party interpretation services due to lack of necessary information.

Additional efforts could be made by publicly accessible variant interpretation resources, such as ClinVar and InterVar, and third-party interpretation services to improve the quality and comprehensibility of genetic information they provide. This could be done by increasing the amount and types of evidence supporting the classification, using easy to understand terms, indicating the strength of the relationship between the variant and the disease, and including finer classification of variants based on their frequencies in populations.

Finally, this study also has its limitations. It is based on the genomic data of five individuals. Although the data are not biased as to pre-selected gene panel or predisposition to false alarms [5], the sample size is small, and not representative of a diverse population. We also only analyzed the false alarms produced by two companies providing DTC genomics service, 23andMe and FamilyTreeDNA. Because the FamilyTreeDNA SNV panel has less coverage in protein coding regions, its false alarm problem is not as significant as 23andMe. Estimates of false alarms may vary according to the number and selection of SNVs on any particular microarray.

## 5. Conclusions

In summary, we found the problem of false alarms due to genotyping errors from consumer array genotyping data (on average >5 per person for 23andMe arrays and 0.2 for the FamilyTreeDNA array) to be highly concerning, assuming there is no change to how DTC companies perform their analysis and no change in how third-party data interpretation services classify variants. Switching to genome sequencing may yield fewer genotyping errors (incorrect results), but will not solve false alarms due to the variant classification problems.

## Figures and Tables

**Table 1:** Observed and expected pathogenic alleles called due to genotyping errors.

Individual ID	Number of Microarray SNVs	Expected Total Wrong Alleles Due to Error	Expected Pathogenic Alleles Due to Error	Observed Pathogenic Alleles Due to Error	Total Observed Pathogenic Alleles
hu85ADFB ^1^	577,585	1674.1	11.4	12	20
hu9AF7CC ^1^	581,079	652.2	5.0	5	13
huC58DAE ^1^	933,148	1084.3	3.8	5	12
huD3FFCB ^1^	930,438	750.3	2.6	8	15
huCDD5EE ^2^	688,413	2269.7	0.2	1	4

^1^ 23andMe DNA data; ^2^ Family Tree DNA data. SNVs—single nucleotide variants.

## Supplementary Materials

The following are available online at https://www.mdpi.com/2075-4426/10/4/187/s1, Note S1: Supplementary Methods, Results and References, Table S1: Counts of genotyping errors of the microarray and sequencing data, Table S2: Details of genotyping errors of the microarray and sequencing data.
