# Supplementary material for: False Alarms in Consumer Genomics Add to Public Fear and Potential Health Care Burden
Source: J Pers Med. 2020 Oct 23;10(4):187. doi: 10.3390/jpm10040187 (PMC7712761; doi:10.3390/jpm10040187)
Supplement: Supplementary file 1 [file jpm-10-00187-s001.zip › Note S1.docx]

**Supplementary Methods**

**Sample Selection**: Five individuals (hu85ADFB, hu9AF7CC, huC58DAE, huD3FFCB, huCDD5EE) were selected from the Harvard Personal Genomics Project [1] as they include microarray genotyping data from 23andMe (hu85ADFB, hu9AF7CC, huC58DAE, huD3FFCB) or Family Tree DNA (huCDD5EE), and whole genome sequencing data (VCF and BAM files) from Veritas Genetics.

**Variant Annotation**: Single-nucleotide variants (SNVs) were annotated with Clinvar [2](downloaded 03/11/2019) and HGMD [3] (professional 2017.2) using the WGSA annotation pipeline.[4] Allele frequencies from various populations were obtained from the PGG.SNV database.[5]

**Genotype Error Estimation**: Microarray genotypes were first compared with genotypes from the VCF file and all chromosome positions that are reported in both files ($N$) were selected. All genotypes that disagreed with each other were manually checked by reviewing the corresponding reads from the BAM files using the integrative genomics viewer, and correct genotypes were determined.[6] Assuming the reported genotypes are correct if microarray genotypes agree with sequence genotype, the number of reference alleles and alternative alleles in the correct genotypes of the $N$ positions were counted as $N_{r}$ and $N_{a}$ respectively. Errors were separated into two types: reference alleles mistakenly called as alternative alleles ($n_{1}$), alternative alleles mistakenly called as other alternative alleles ($n_{2}$), and alternative alleles mistakenly called as reference alleles ($n_{3}$). The corresponding error rates were calculated as $r_{1}=n_{1}/N_{r}$ $r_{2}=n_{2}/N_{a}$, and $r_{3}=n_{3}/N_{a}$.

**Expected Pathogenic Genotypes Due to Genotyping Error**: Assuming all microarray genotypes that agree with genotypes reported in VCF files, and those not reported in the corresponding VCF files are correct, given the error rates $r_{1}=1\times{10}^{-3}$ (ref to alt), $r_{2}=6\times{10}^{-5}$ (alt to alt), and $r_{3}=4\times{10}^{-4}$ (alt to ref), we can produce a list of all possible wrong genotypes with corresponding probabilities, for all chromosome positions reported in the microarray data. The list of SNVs were annotated and the summation of the probabilities of those genotypes annotated as Pathogenic or Likely Pathogenic by Clinvar were deemed the expected pathogenic genotypes due to genotyping error. These calculations were conducted for each individual separately.

**Supplementary Results**

For the array data, 4 individuals have complete 23andMe genotypes (2 have ~580,000 SNVs and 2 have ~930,000 SNVs) and 1 has FamilyTreeDNA genotypes (688,413 SNVs) (Supplementary Table 1). Comparing the array genotypes with the corresponding sequencing results (vcf files), a total of 1,414,206 (263,802 to 445,043 per person) SNV genotypes were reported by both the array and vcf, and among them a total of 4,979 disagreements (363 to 2,139 per person) were found between the array reports and sequencing reports (Supplementary Table 1). We manually checked all 4,979 disagreements by reviewing the corresponding raw sequencing data (BAM files). For array data, we found the average error rate of a reference allele called as an alternative allele to be $1\times{10}^{-3}$, an alternative allele called as another alternative allele to be $6\times{10}^{-5}$, and an alternative allele called as the reference allele to be $4\times{10}^{-4}$. Based on the error rates, we estimate that on average an individual has 1,286 wrong alternative alleles called (652 to 2,270 per person) (Supplementary Table 1). Additionally, we investigated to what extent wrong genotypes may raise false alarms for disease susceptibility. As the Clinvar database is popularly used for interpreting the disease impact of a variant, we used it to determine whether a particular SNV will be annotated as pathogenic or likely pathogenic. We estimate that on average a person has 5.7 or 0.2 wrongly called pathogenic SNVs if using a 23andMe array or FamilyTreeDNA array respectively. In reality, the four individuals with 23andMe array data have an average of 7.5 pathogenic SNVs due to wrongly called alternative alleles (false positives), and an average of 7.5 true pathogenic SNVs with correct genotypes (Supplementary Table 1). On the other hand, the individual with FamilyTreeDNA data has 1 false positive pathogenic SNV and 3 true pathogenic SNVs (Supplementary Table 1).

**Supplementary Reference:**

1. The Harvard Personal Genome Project (PGP) – enabling participant-driven science. Available at: https://pgp.med.harvard.edu/. (Accessed: 25th June 2019)

2. Landrum MJ, Lee JM, Benson M, Brown GR, Chao C, Chitipiralla S, Gu B, Hart J, Hoffman D, Jang W, Karapetyan K, Katz K, Liu C, Maddipatla Z, Malheiro A, McDaniel K, Ovetsky M, Riley G, Zhou G, Holmes JB, Kattman BL, Maglott DR. ClinVar: improving access to variant interpretations and supporting evidence. Nucleic Acids Res. 2018; 46:D1062–D1067.

3. Stenson PD, Mort M, Ball EV, Shaw K, Phillips A, Cooper DN. The Human Gene Mutation Database: building a comprehensive mutation repository for clinical and molecular genetics, diagnostic testing and personalized genomic medicine. Human Genetics 2014; 133:1–9.

4. Liu X, White S, Peng B, Johnson AD, Brody JA, Li AH, Huang Z, Carroll A, Wei P, Gibbs R, Klein RJ, Boerwinkle E. WGSA: an annotation pipeline for human genome sequencing studies. J Med Genet 2016; 53:111–112.

5. Zhang C, Gao Y, Ning Z, Lu Y, Zhang X, Liu J, Xie B, Xue Z, Wang X, Yuan K, Ge X, Pan Y, Liu C, Tian L, Wang Y, Lu D, Hoh BP, Xu S. PGG.SNV: understanding the evolutionary and medical implications of human single nucleotide variations in diverse populations. Genome Biol 2019; 20:215.

6. Robinson JT, Thorvaldsdóttir H, Winckler W, Guttman M, Lander ES, Getz G, Mesirov JP. Integrative genomics viewer. Nat Biotechnol 2011; 29:24-6.
